# Robotic Removal of Spilled Gallstones After Laparoscopic Cholecystectomy: Resolving Chronic Abdominal Pain Using New Technology

**DOI:** 10.7759/cureus.64618

**Published:** 2024-07-15

**Authors:** Alexander H Vu, Jacqueline E Woo, Jessica Chiang, Michael Timoney

**Affiliations:** 1 General Surgery, New York University (NYU) Langone, Brooklyn, USA; 2 Institute of Human Nutrition, Columbia University College of Physicians and Surgeons, New York, USA

**Keywords:** lap chole, lap cholecystectomy, robotic surgical procedures, gallstone, surgery

## Abstract

Spilled gallstones during laparoscopic cholecystectomy can potentially lead to serious complications in patients. We present a case of a patient with gallstone spillage during cholecystectomy who was found years later to have gallstones stuck in a difficult location, requiring robotic surgery. A robotic approach allows for greater visual angles compared to conventional laparoscopy. The patient tolerated the robotic procedure successfully, and no patient symptoms were reported during follow-up. This case addresses retained gallstones for difficult anatomical positions and confirms that a robotic abdominal approach is a safe, minimally invasive option.

## Introduction

Gallstone spillage during laparoscopic cholecystectomy is a frequent occurrence, up to 30%, causing frustration for surgeons and potentially serious complications in patients [1]. Robotic platforms offer advantages over conventional laparoscopy, including the ability to obtain visual angles. We describe a case of a patient with a common problem - gallstone spillage during cholecystectomy - who was found years later to have gallstones stuck in a location that was difficult to reach, requiring an innovative use of robotic surgery. We review the literature on the pathophysiology, diagnosis, and treatment of spilled gallstones. To our knowledge, this is the only reported case of addressing dropped gallstones and subsequent abscesses using a robotic abdominal approach.

Pathophysiology

Spilled gallstones, also known as dropped gallstones or retained gallstones, are usually noted at the time of surgery, but if they go unrecognized, they can lead to an uncommon postoperative, potentially morbid complication after laparoscopic cholecystectomy. The unnoticed, spilled intraperitoneal gallstone can be disseminated anywhere in the abdomen or pelvis due to pneumoperitoneum, irrigation, and laparoscopic attempts to remove the perforated gallbladder [1]. The spilled gallstone then acts as a nidus of infection as bacteria form a glycocalyx biofilm on the stone’s surface, leading to inflammation and granulation [2]. Initially, intraperitoneal spilled gallstones incite granuloma formation, which is followed by the formation of abscesses, sinus tracts, and fistulas, and, in some cases, systemic symptoms [1]. Possible areas affected depend on where the gallstone had been spilled, either from the port site or the perihepatic/surgical spaces; however, as mentioned before, the retained gallstone can translocate anywhere in the abdominopelvic spaces [1]. Pigment stones demonstrate viable enteric and non-enteric organisms on electron microscopy and, therefore, lead to a higher risk of intra-abdominal infection [3].

Epidemiology

The incidence of perforation during laparoscopic cholecystectomy is as high as 40% [3]. The majority of perforations occur during dissecting the gallbladder off the liver bed and during extraction of the gallbladder through the trocar site [4]. Risk factors for gallbladder perforation include distended gallbladder, acute or chronic cholecystitis with wall thickening greater than 7 mm, difficult dissection, prior laparotomy, male sex, greater weight, older age, presence of adhesions, and surgeon inexperience [3-4]. Several large systematic reviews have recently described the incidence of spilled gallstones. The study by Gavriilidis et al. estimates that the rate of spilled gallstones is 16% and that 16 to 50% of these gallstones are not retrieved [2]. Demirbas et al. reported that the incidence of spilled stones ranges from 6% to 40% [4]. The complication rate from spilled gallstones is reported to be between 0.3% and 19% [2,5]. Complications include intrabdominal abscesses, abdominal wall abscesses, and retroperitoneal abscesses [5]. Risk factors for complications include advanced age, spillage of infected bile, pigment stones, perihepatic location, more than 15 spilled gallstones, gallstones greater than 1.5 cm in size, and male sex [4-6]. The majority of complications arise from intra-abdominal abscess formation. The incidence is 0.08% to 1.5%, while retained gallstones affecting the thoracic cavity are estimated to have an incidence of 0.08% to 0.3% [7].

Diagnosis

If not discovered immediately, retained gallstones are frequently diagnosed long after the index procedure, with a median interval time to diagnosis of five months, with some even describing up to 20 years before diagnosis. These discoveries are often made in the context of recurrent intra-abdominal abscess formation [1]. Workup is primarily image-based and can include ultrasound, computed tomography (CT), and magnetic resonance imaging (MRI). In the US, stones are hyperechoic with acoustic shadowing [3,8]. On CT, they may appear either hypodense or hyperdense [3]. On MRI, they can appear as signal voids or hyperintense on T1-weighted sequences and appear hypointense on T2-weighted sequences; however, surrounding granulomatous reactions can obscure radiographic diagnosis [1,3,8]. Reddy et al. argue that MRI can provide more anatomic and functional information and may be a more sensitive and useful modality than CT in picking up non-calcified gallstones and identifying postoperative complications after laparoscopic cholecystectomy [5]. Spilled gallstones have also been reported to radiographically masquerade as malignant peritoneal seeding, sarcomas, and primary tumors [1,3].

Management of symptomatic spilled gallstones

First, prevention includes preventing spilled gallstones with careful dissection and aspiration of bile from a tense gallbladder [1]. If spillage occurs, an immediate attempt at removal remains paramount to preventing complications. To aid in laparoscopic removal, one may place additional ports, use a 30° scope, or use copious irrigation [1]. Furthermore, management of spilled gallstones is dictated by location: if in the pelvis, gynecologic or urologic intervention may be required. If in the thorax, thoracic surgical intervention may be required. Percutaneous drainage and antibiotics may be options, but definitive management via stone extraction (laparoscopic vs. open) remains the recommended approach [3]. Large stones can be fragmented using ultrasound lithotripsy and rigid endoscopy [1]. When all of these attempts are unsuccessful, repeat laparoscopy or laparotomy may be considered [1]. Gavriilidis et al. report that a majority of spilled gallstone cases ultimately underwent open abdominal exploration [2]. There is a lack of data concerning the success rate of the laparoscopic approach, the conversion rate from laparoscopic to open, and the complications of a laparoscopic approach to retaining spilled gallstones in the literature.

## Case presentation

We present a case of a 59-year-old male with no significant past medical or surgical history, who originally had undergone an elective laparoscopic cholecystectomy by another outside hospital surgeon seven years prior for biliary colic, complicated by a seven-year saga of chronic abdominal pain due spilled gallstones. The procedure was remarkable for perforated cholecystitis and spillage of biliary contents that were reportedly suctioned and removed.

**Figure 1:**
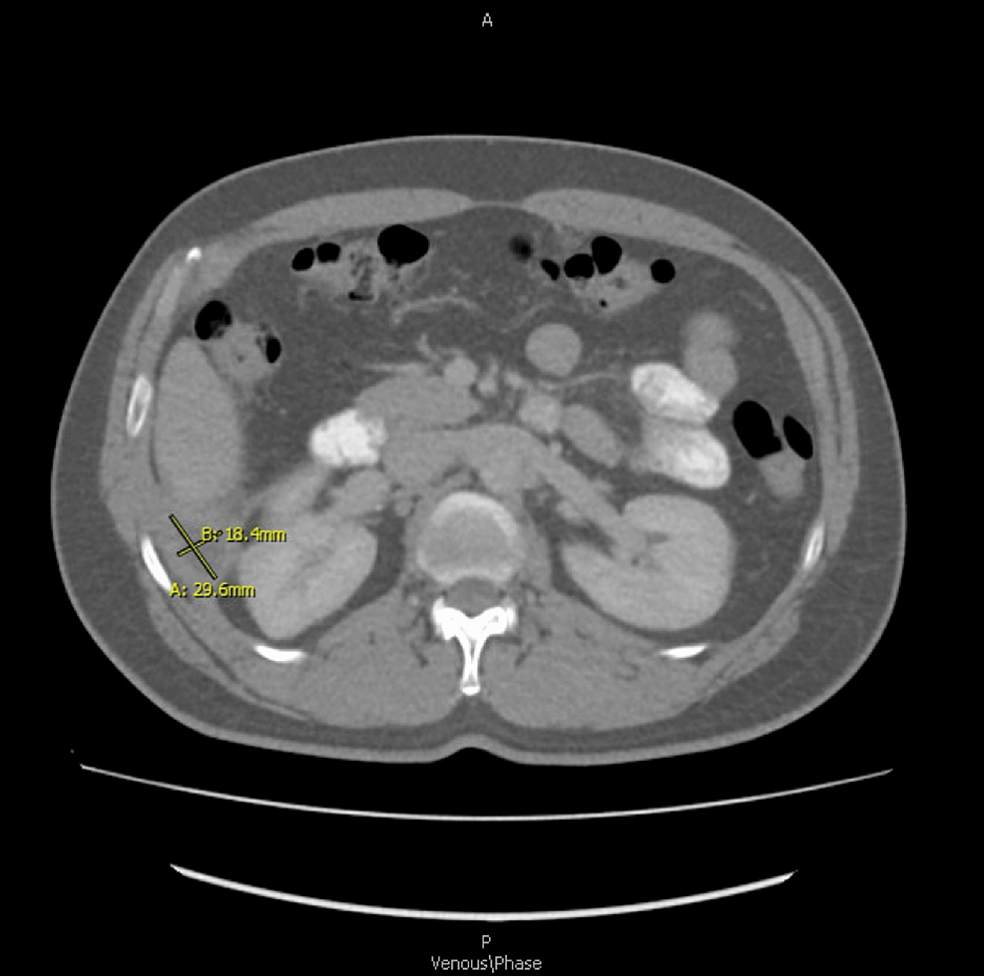
Equivocal CTAP imaging, axial (one year after surgery) representative of infra-hepatic abscess vs. potential retained spilled stones. Radiology at this time felt the findings were more consistent with abscesses. CTAP: computed tomography arterial portography

Since discharge, the patient had months of intermittent episodes of subjective fevers and epigastric pain. One year later, he was then admitted to the surgical floor for worsening epigastric pain after CT imaging demonstrated a possible hypodense lesion concerning infrahepatic abscess vs. spilled gallstone. The interpreting radiologist felt this was more consistent with a walled-off abscess (Figure 1). Thus, the patient was admitted and underwent interventional radiology (IR) CT-guided aspiration of 4 mL of cloudy effluent, cultures remarkable for *Escherichia coli*, and ultimately discharged three days later on a short course of cefpodoxime and metronidazole.

Three years later, the pain persisted and the patient sought a second opinion with a gastroenterologist, who performed esophagogastroduodenoscopy and colonoscopy, which were ultimately remarkable for diverticulosis and *Helicobacter pylori* gastritis, and thus treated. During this time, the patient also underwent an additional IR drainage for an increased-sized recurrent, infrahepatic abscess, remarkable for *E. coli*.

**Figure 2:**
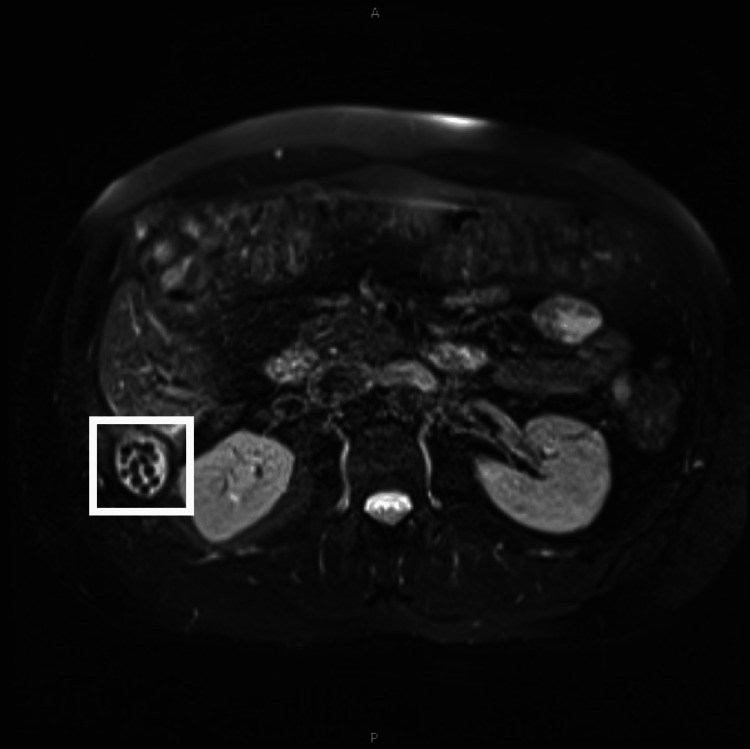
MRCP in seven years after surgery that visualizes more clearly retained spilled gallstones. The area of interest is marked. MRCP: magnetic resonance cholangiopancreatography

About seven years later, the patient still endorsed chronic right-sided discomfort but was otherwise non-peritoneal, and at this time, nonoperative management was felt to be appropriate. Given the recurrence and lower suspicion of retained stone, gastroenterology was re-consulted outpatient for potential occult liver/colon cancer as an etiology. During this time, the patient was retreated for *H. pylori* twice and also reported that his abdominal pain had radiated to his flank, prompting a urology workup, remarkable for microscopic hematuria on urinalysis and left kidney cyst; however, it was deemed unlikely the culprit for symptoms. He ultimately underwent an MRI for a malignancy work-up. However, the scan was remarkable for numerous spilled stones and a 2.6 × 2.1 × 2.3 cm collection in the posterior right-upper quadrant abdomen (Figure 2). The patient visited our surgical outpatient clinic, and perioperatively, a robotic approach was considered, given the difficult positioning of the patient's abscess in Morison’s pouch.

**Figure 3:**
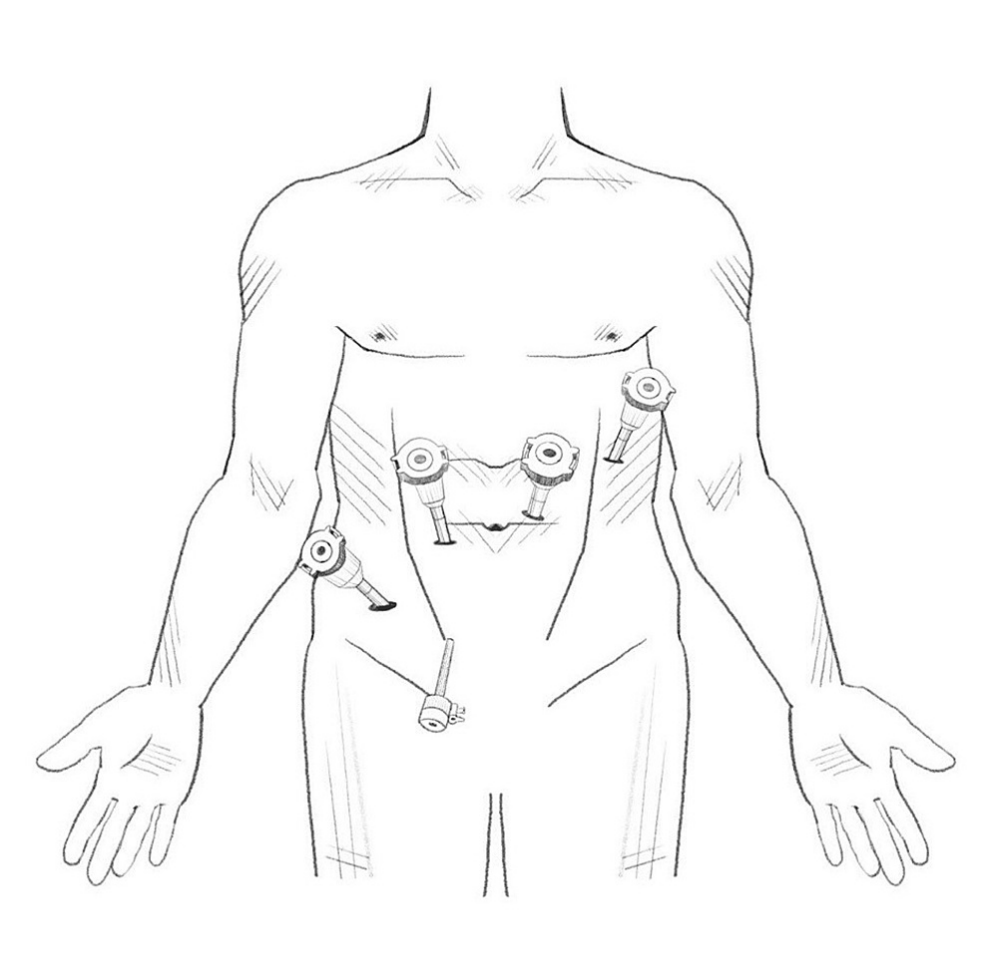
An illustration of robotic 8-mm port placements with arms 1, 2, 3, and 4 with fenestrated bipolar, 30° camera, hook cautery, and tip-up grasper, respectively, with a 5-mm port placement as an assistant port by Susanna Yun, BFA.

**Figure 4:**
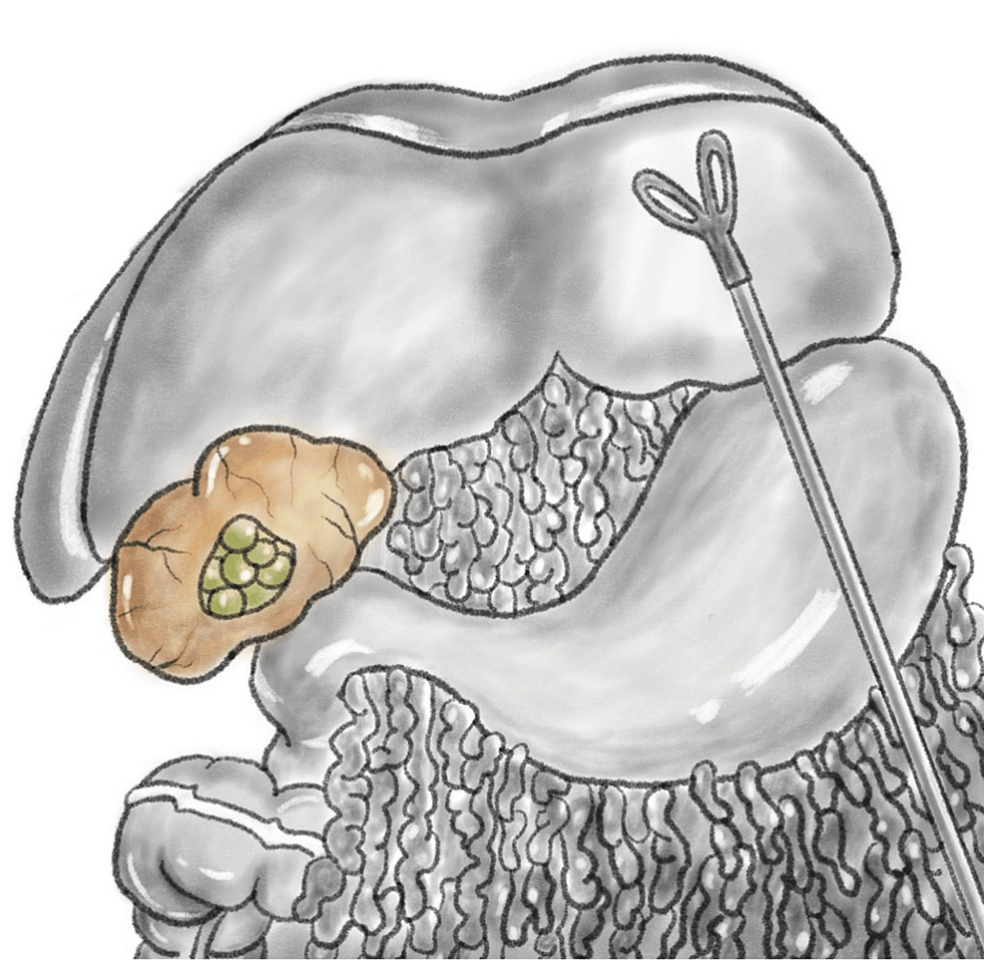
An illustration of the anatomically difficult, acute angle of Morison’s pouch with abscess cavity by Susanna Yun, BFA.

The patient was brought to the operating room and placed in the supine position on the operating table. A roll was placed under his right flank to corkscrew the patient anteriorly on that side. After the induction of general endotracheal anesthesia, the abdomen was prepped and draped in the standard surgical manner. The abdomen was insufflated via Veress through the umbilicus. Initial trocar placement was unremarkable, with ×4 8 mm robotic ports with an additional 5-mm assist port (Figure 3). Anterior omental adhesions were encountered and taken down, and Da Vinci Xi was docked. Robotic ports 1, 2, 3, and 4 were equipped with a fenestrated bipolar, 30° camera, hook cautery, and tip-up grasper, respectively (Figure 3). The right colon and the transverse colon were plastered to the liver and were carefully freed using a combination of robotic sharp and blunt dissection. Eventually, we were able to free the space of Morison's pouch under the liver at the junction to Gerota's fascia, which was inflamed from the chronic infection. Using cautery, a window into Morison's pouch was created between Gerota's fascia and the liver. A scant amount of pus emanated from a pocket in Morison's pouch and ultimately revealed multiple retained stones (Figure 4). Approximately 20 small stones varying from sub-centimeter to 1 cm were extracted into EndoCatch bags. Morison’s pouch was then washed out and irrigated, and hemostasis was excellent without any injury to the bowel. Jackson-Pratt drain was placed, and sterile dressings were applied. Operative time was two hours, and the estimated blood loss was 50 cc. The patient tolerated the procedure well and was sent to recovery after extubation. The postoperative hospital stay was two days. The patient was seen outpatient one week later for drain removal, and the pathology was remarkable for *E. coli* abscess cavity and pigment-type gallstones. The patient, at a one-year postoperative visit, reported complete resolution of abdominal pain.

## Discussion

Many aspects of this case are consistent with the typical epidemiology and presentation of spilled gallstones. Risk factors specific to this case include being male, advanced age, perforated cholecystitis, and spillage of biliary contents. Consistent with the most common complication of spilled gallstones, the complication this patient had was an intra-abdominal abscess in the most commonly affected space (infrahepatic/Morison’s pouch) [2]. Additionally, at some point, gastroenterology was consulted for concern for malignancy or occult liver lesions, which spilled gallstones often mimic radiographically [1,3].

There are several points of discussion regarding this case. First, at the index operation, it was important to reduce the likelihood of retained spilled gallstones. This may include the use of copious irrigation or an additional 5-mm port placement to aid in manipulating surrounding organs and retrieval of stones. Second, MRI should have been considered early in the workup of this man’s pain syndrome, especially since the first postoperative CT scan was indeterminate in diagnosing spilled gallstones. MRI, as mentioned before, may be more sensitive to identifying non-calcified gallstones [5]. Third, we demonstrate the ability of a robotic approach to safely manage and address this difficult-to-reach anatomical space. As Gavriilidis et al. reported in their large systematic review in 2022, the overwhelming majority of retained gallstone cases that resulted in complications ultimately underwent open abdominal exploration, the implications of which result in a longer length of stay and higher risk of incisional hernia [2]. Presumably, the need for conversion to open may involve the extensive need for lysis of adhesions and difficulty visualizing Morison’s pouch and other difficult angles of view. Chronically retained gallstones and resultant inflammation, coupled with the difficult anatomical positioning of Morison’s pouch, may make a laparoscopic approach to retained gallstones prohibitively difficult. A robotic approach allows for greater degrees of freedom and excellent optics to safely address densely adherent and inflamed tissue in Morison’s pouch. When anticipating extensive adhesions in difficult anatomical positions, a robotic approach may provide a safe, minimally invasive option.

## Conclusions

Our case highlights the importance of addressing spilled gallstones promptly to prevent potential complications. The patient’s prolonged course of chronic abdominal pain, recurrent abscesses, and diagnostic challenges underscore the need for vigilant follow-up and consideration of advanced imaging modalities such as MRI in cases of persistent symptoms. This report presents the first documented case of successful retrieval of retained gallstones from a challenging anatomical location using a robotic abdominal approach, emphasizing the feasibility and safety of this minimally invasive technique. The use of robotics allowed for precise visualization and maneuverability in Morison's pouch, providing a valuable alternative to traditional open exploration, particularly in cases with difficult angles and extensive adhesions. This novel approach demonstrates the potential of robotics in managing complications related to spilled gallstones and offers a promising avenue for further exploration in future challenging scenarios.

## References

[1] Ramamurthy NK, Rudralingam V, Martin DF, Galloway SW, Sukumar SA (2013). Out of sight but kept in mind: complications and imitations of dropped gallstones. AJR Am J Roentgenol.

[2] Gavriilidis P, Catena F, de'Angelis G, de'Angelis N (2022). Consequences of the spilled gallstones during laparoscopic cholecystectomy: a systematic review. World J Emerg Surg.

[3] Jabbari Nooghabi A, Hassanpour M, Jangjoo A (2016). Consequences of lost gallstones during laparoscopic cholecystectomy: a review article. Surg Laparosc Endosc Percutan Tech.

[4] Demirbas BT, Gulluoglu BM, Aktan AO (2015). Retained abdominal gallstones after laparoscopic cholecystectomy: a systematic review. Surg Laparosc Endosc Percutan Tech.

[5] Reddy S, Lopes Vendrami C, Mittal P, Borhani AA, Moreno CC, Miller FH (2021). MRI evaluation of bile duct injuries and other post-cholecystectomy complications. Abdom Radiol (NY).

[6] Brockmann JG, Kocher T, Senninger NJ, Schürmann GM (2002). Complications due to gallstones lost during laparoscopic cholecystectomy. Surg Endosc.

[7] Perrone G, Giuffrida M, Tarasconi A, Bonati E, Catena F (2021). Thoracic complications from retained abdominal gallstones after laparoscopic cholecystectomy: is it always mandatory a thoracic approach?. Ulus Travma Acil Cerrahi Derg.

[8] Nayak L, Menias CO, Gayer G (2013). Dropped gallstones: spectrum of imaging findings, complications and diagnostic pitfalls. Br J Radiol.

